# Experimental Advances in Nanoparticle-Driven Stabilization of Liquid-Crystalline Blue Phases and Twist-Grain Boundary Phases

**DOI:** 10.3390/nano11112968

**Published:** 2021-11-05

**Authors:** George Cordoyiannis, Marta Lavrič, Vasileios Tzitzios, Maja Trček, Ioannis Lelidis, George Nounesis, Samo Kralj, Jan Thoen, Zdravko Kutnjak

**Affiliations:** 1 Condensed Matter Physics Department, Jožef Stefan Institute, 1000 Ljubljana, Slovenia; marta.lavric@ijs.si (M.L.); maja.trcek@ijs.si (M.T.); zdravko.kutnjak@ijs.si (Z.K.); 2 Faculty of Mechanical Engineering, Czech Technical University in Prague, 16600 Prague 6, Czech Republic; 3 Institute of Nanoscience and Nanotechnology, National Centre for Scientific Research “Demokritos”, Aghia Paraskevi, 15310 Athens, Greece; v.tzitzios@inn.demokritos.gr; 4 Faculty of Physics, National and Kapodistrian University of Athens, Zografou, 15784 Athens, Greece; ilelidis@phys.uoa.gr; 5 Institute of Nuclear and Radiological Sciences and Technology, National Centre for Scientific Research “Demokritos”, Aghia Paraskevi, 15310 Athens, Greece; nounesis@rrp.demokritos.gr; 6 Faculty of Natural Sciences, University of Maribor, 2000 Maribor, Slovenia; samo.kralj@um.si; 7 Department of Physics and Astronomy, KU Leuven, 3001 Leuven, Belgium; jan.thoen@kuleuven.be

**Keywords:** liquid crystals, nanoparticles, quantum dots, reduced-graphene oxide, calorimetry, microscopy, blue phases, twist-grain boundary phases, disclination lines, screw dislocations

## Abstract

Recent advances in experimental studies of nanoparticle-driven stabilization of chiral liquid-crystalline phases are highlighted. The stabilization is achieved via the nanoparticles’ assembly in the defect lattices of the soft liquid-crystalline hosts. This is of significant importance for understanding the interactions of nanoparticles with topological defects and for envisioned technological applications. We demonstrate that blue phases are stabilized and twist-grain boundary phases are induced by dispersing surface-functionalized CdSSe quantum dots, spherical Au nanoparticles, as well as MoS_2_ nanoplatelets and reduced-graphene oxide nanosheets in chiral liquid crystals. Phase diagrams are shown based on calorimetric and optical measurements. Our findings related to the role of the nanoparticle core composition, size, shape, and surface coating on the stabilization effect are presented, followed by an overview of and comparison with other related studies in the literature. Moreover, the key points of the underlying mechanisms are summarized and prospects in the field are briefly discussed.

## 1. Introduction

Liquid crystals (LCs) are soft materials exhibiting many intermediate phases, the so-called mesophases, with structures in between the high-symmetry disordered liquid and the low-symmetry ordered crystal phases. Upon reducing the temperature, they undergo several symmetry-braking phase transitions and gradually acquire orientational and partial positional order. The first liquid-crystalline material, cholesteryl benzoate, was experimentally discovered by the Austrian botanist F. Reinitzer towards the end of the 19th century [1]. However, it was exactly one century ago when G. Friedel [2] contributed the nomenclature of the first liquid-crystalline mesophases (nematic, smectic) and about half a century ago when liquid crystals found their inroads into optical display applications [3].

LCs respond strongly to even weak perturbations due to their soft, fluid-like character. In the past, the feature mentioned above as well as the various types of transitions occurring between mesophases, led to the choice of LCs and nanoparticles (NPs) mixtures as model systems for testing the disorder and confinement effects upon phase transitions and critical phenomena [4,5,6,7,8,9,10]. Surface-wetting and finite-size effects have also been investigated experimentally and theoretically [11,12]. More recently, LCs have been exploited as soft matrices for hosting regular templates of colloids [13,14,15] and NPs [16,17,18,19]. The anisotropy in the optical and dielectric properties of LCs combined with the dispersion of NPs, which contribute additional functionalities to the resulting soft nanocomposites, could trigger novel applications of LCs in the fields of micro-, bio-, sensing-, and nanotechnologies [20,21].

In some chiral LCs compounds, the competition between the packing topology and the chirality generates peculiar structures stabilized by lines of topological defects in short temperature regimes. Typical examples of such structures are the liquid-crystalline blue phases (BPs) and twist-grain boundary phases (TGBs). BPs are characterized by lattices formed by disclination lines, whereas TGBs by screw dislocations. Disclinations are defects in the orientational order; they appear in regions where the local nematic director cannot be uniquely defined and are characterized by winding numbers 1/2 or −1/2. Screw dislocations are defects in the translational order; in these points the smectic order parameter is melted. The general background and the main features of BPs and TGBs are introduced in the following section. The subsequent sections demonstrate how NPs assemble within lattices of topological defects and increase the temperature stability range of these phases or even induce them when they do not exist (or exist in a metastable state) in the pure LCs.

## 2. Background of Liquid-Crystalline Blue Phases and Twist-Grain Boundary Phases

### 2.1. Blue Phases

BPs were present (albeit unknown at that time) in cholesteryl benzoate, since Reinitzer noticed already some reflections of green and blue [1]. However, they were essentially brought to the attention of the scientific community much later [22], and the term ‘blue phase’ was introduced in a study of cholesteryl compounds by Coates and Gray [23]. On the basis of elasticity, Meiboom et al. [24] introduced the free energy expression for BPs and proposed the structure of interlaced double-twist tubes as a potential, energy-favored configuration.

**Figure 1:**
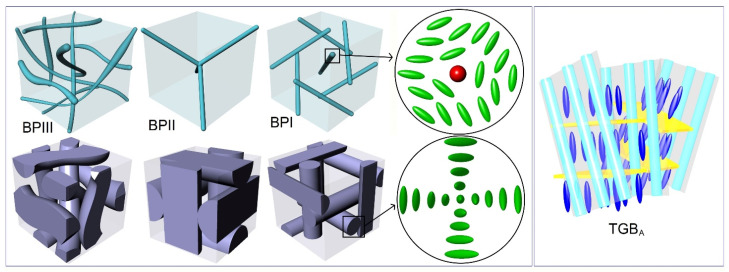
Left panel: the structure of BPIII, BPII, and BPI is shown from the left to right. For BPs, the top images show the network of disclination lines, being amorphous for BPIII, simple cubic for BPII, and body-centered cubic for BPI. The bottom images show the shapes of the double-twist cylinders. The magnifications indicate the LC molecular orientation in the vicinity of a −1/2 disclination line (**top**) and along a cut of the double-twist cylinder (**bottom**). Right panel: the TGB_A_ structure is depicted; screw dislocations along the grain boundaries separate slabs of layered SmA order.

BPs are thermodynamically stable in a short (typically a couple of K) temperature range between the isotropic and the cholesteric phases of some strongly-chiral LCs [25]. Three types of BPs have been found and characterized as blue phase III (BPIII), blue phase II (BPII), and blue phase I (BPI) in order of decreasing temperature. Numerous studies have focused on deciphering their structure, yielding a macroscopically amorphous network of disclination lines for BPIII [26], changing to a three-dimensional simple cubic for BPII and a body-centered cubic lattice for BPI [27,28]. In the case of BPIII and BPII, the disclination lines are entangled and interconnected; on the contrary, in the case of BPI, they do not intersect [29,30]. Between the disclination lines, there exists liquid-crystalline type of order; the LC molecules are oriented along double-twisted cylinders as depicted in Figure 1. In the middle of these cylinders the director of LC molecular orientation is parallel to the cylinder axis, whereas it progressively changes from −45° to 45° at the periphery.

Until the end of the 20th century, the scientific interest in BPs was limited to fundamental studies, such as investigating phase diagrams as a function of chirality and the critical behavior [31,32,33,34,35]. However, the ascertainment that the three-dimensional photonic bandgap structures of BPII and BPI exhibit periodicities in visible wavelengths could open new pathways towards applications in photonics, as pointed by Etchegoin [36]. Soon after, Cao et al. [37] demonstrated the potential of BP structures for applications such as tunable soft lasers. In classical cholesteric LCs, lasing is observed only along one dimension, whereas in BPs it is possible in three dimensions. Therefore, LCs could be viewed as candidates for photonic bandgap applications. These findings evoked a major revival of the research interest in studying and stabilizing these phases over wider temperature ranges [38].

### 2.2. Twist-Grain Boundary Phases

TGBs often appear between the cholesteric and the smectic phases of strongly chiral LCs. Their existence was conceptualized by de Gennes [39] and the theoretical formulation completed by Renn and Lubensky [40]. TGBs comprise the liquid-crystalline analogue of the Shubnikov phase, characterized by Abrikosov flux vortices in type-II superconductors. The isomorphism among LCs and superconductors’ phases reads as follows: cholesteric (N*)—metal, twisted chiral-line (N_L_*)—Abrikosov vortex liquid, twist-grain boundary A (TGB_A_)—Abrikosov vortex lattice, smectic A (SmA)—Meissner phase. The verification of the existence of TGBs in pure LC compounds and LC mixtures came out in a sequence of experimental studies by Goodby et al. [41], Lavrentovich et al. [42], and Nguyen et al. [43]. The thermal signature, the structure, and the optical textures of the TGB_A_ phase have been clearly identified [44,45,46,47,48]. TGB_A_ consists of slabs of SmA-type of order separated by one-dimensional lattices of screw dislocations along grain boundaries, as depicted in Figure 1. This defect lattice is pinned in the case of TGB_A_, whereas it oscillates in the case of N_L_* phase that exhibits a short-range TGB order [49]. Distinguishing the small thermal signatures and the structural differences of N_L_* and TGB_A_ phases ultimately requires accurate high-resolution calorimetric and small-angle X-ray measurements [17,46]. Note that the twist-grain boundary C or C* (TGB_C_, TGB_C_*) phases can also be observed; in this case, smectic-C- or chiral smectic-C*-type of order exists along the slabs [43,50,51,52,53].

This review presents recent experimental efforts on stabilizing both BPs and TGBs by dispersing either spherical or anisotropic NPs in chiral LCs. By choosing the appropriate core composition, size, geometry, and surface chemistry of NPs, the latter can assemble within the defect lattices and increase the stability of these phases over broader temperature ranges. It is shown how calorimetric and optical methods are combined in order to sense the BP and TGB stabilization when dispersing small CdSSe quantum dots, spherical Au nanoparticles, and larger reduced-graphene oxide nanosheets in chiral LCs. The results are obtained on two chiral LCs that exhibit all three BPs, howbeit no stable TGB_A_ phase. The similarities and differences in the stabilization effect are discussed with respect to the size, shape, core composition, and surface functionalization of NPs. Afterwards, the mechanisms that govern the stabilization are briefly addressed. Further on, we refer to the main findings of other related studies in literature, using particles with sizes ranging from the nano- to the microscale. Our review will be concluded by summarizing the advances in the field and remaining open questions.

## 3. Experimental Results in Blue Phase and Twist-Grain Boundary Phase Stabilization

### 3.1. Blue Phase Stabilization by Inclusions: From Polymers and Dopants to Nanoparticles

The first robust strategy for stabilizing BPs by inclusions was introduced by Kikuchi et al. [38] based on bi-continuous phase separation phenomena in a polymer-doped LC mixture. BPI was stabilized over a wide temperature range via the alleged assembly of the polymer chains in the disclination lines. Soon after, doping of chiral LC with non-chiral bent shape molecules has also been proposed as a means of BP stabilization by Nakata et al. [54]. NP-driven stabilization was first reported by Yoshida et al. [55], who dispersed Au NPs with a diameter of 3.7 nm in a multi-component LC mixture. Finally, stabilization in single LC compounds was reported by Karatairi et al. [56] and Cordoyiannis et al. [57], by dispersing 3.5 nm CdSe quantum dots surface-functionalized with OA and TOP in *S*-(+)-4-(2′-methylbutyl) phenyl-4′-*n*-octylbiphenyl-4 carboxylate (CE8), and in S-(+)-4-(2-methylbutyl) phenyl-4-decyloxybenzoate (CE6), respectively. In both cases [56,57], the CdSe quantum dots essentially broadened (almost ten-fold) the temperature range of BPIII at the expense of BPII (gradually disappeared), whereas BPI was mildly affected. It is worth mentioning that, despite their similar acronyms, CE6 and CE8 do not belong to the same homologues series, thus, do not have similar chemical formulas. From these studies [55,56,57] it became evident that NPs can be utilized to stabilize BPs over wider temperature ranges with respect to pure LCs. Apart from the stabilization effect, the dispersion of proper NPs in LCs can additionally improve the LC electro-optical performance. For example, increased dielectric anisotropy and enhanced Kerr effect, resulting in a low-voltage and hysteresis-free fast switching, have been demonstrated for BPI of an LC doped with BaTiO_3_ NPs [58].

As mentioned above, in the case of polymer-induced widening of the BPI range, the stabilization effect has been attributed to the aggregation of polymer chains along the disclination lines [38]. The crucial point is that the cores of defects with essentially melted liquid-crystalline order, i.e., the disclination lines, are partially replaced by polymer chains. The replacement of the energy-costly defect cores by polymer chains reduces the free energy of the LC and polymer composite, leading to a more stable BP structure. This has been later on extended in the case of NP-driven stabilization, as discussed in the following sections.

### 3.2. Choice of Materials and Methods for Systematically Exploring the Stabilization Effect

For the vast majority of the experimental results overviewed in the following sections, various types of spherical and anisotropic NPs have been dispersed in CE8. The thermal signatures of BPIII, BPII, and BPI are present in pure CE8, within a total temperature range of ~5 K [56], making it an ideal compound for exploring the impact of inclusions on all three BPs. In addition, calorimetric measurements indicate the existence of a metastable TGB_A_ phase, along the low-temperature wing of the first order N*-SmA transition, due to the apparent proximity (of pure CE8) to a triple point of coexisting N*, TGB_A_, and SmA phases [59]. Observations of optical textures confirm that this metastable TGB_A_ order can be stabilized when CE8 is confined between treated glass surfaces; the stabilization depends on the glass surface-anchoring (planar, homeotropic, rubbed or untreated glass) and the cell thickness (spacers of 10 or 20 μm) [60].

NPs of different inorganic core composition, size, shape, and surface chemistry have been synthesized at the National Centre for Scientific Research “Demokritos” (Greece). Thermolytic approaches have been followed in high boiling point organic solvents, under the presence of appropriate capping agents depending on the nature of inorganic material [61]. Experimental results on mixtures of CE8 with the following NPs are overviewed: (a) CdSSe quantum dots with an average diameter of 3.4 nm, surface grafted by oleyl amine (OA) and trioctyl phosphine (TOP) molecules, (b) spherical Au NPs with an average diameter of 10 nm and OA coating, (c) MoS_2_ nanoplatelets with an average size of 10 nm, consisting of two to four layers and surface-functionalized with OA, (d) reduced-graphene oxide (r-GO) nanosheets with an average size of 50 nm and OA coating, and (e) r-GO nanosheets with an average size of 50 nm, decorated with CoPt NPs that enhance the OA coverage. The concentration *χ* is defined in all cases as the mass of NPs over the total sample mass. For preparing the mixtures, the LC mass is weighed on a balance with accuracy of ±0.05 mg. Appropriate quantities of NPs solutions, after mild sonication, are added by high-precision pipettes and the components are slowly heated and magnetically stirred to achieve homogeneous dispersion of the NPs in the LC host.

Measurements have been performed by combining high-resolution ac calorimetry and polarizing optical microscopy at the Jožef Stefan Institute (Slovenia) and at the University of Athens (Greece), respectively. The combination of these two methods, providing both the temperature dependence of heat capacity and the optical textures upon heating and cooling, confirms the thermal stability of the observed phases and excludes the possibility of apparent, surface-induced [60] or super-cooled [62] phases. Details regarding the standard protocols for the mixtures’ preparation can be found in previous studies [56,57,63]. A thorough description and technical details of ac calorimetry can be found elsewhere [64,65].

### 3.3. Blue Phase and Twist-Grain Boundary Phase Stabilization in Liquid Crystals CE8 and CE6 Induced by Spherical Nanoparticles

The experimental proof of NP-induced BP stabilization [55,56,57] has been attributed to the assembly of the former in the defect cores. The so-called defect core replacement (DCR) mechanism has been proposed [66], based on the initial assumptions of Kikuchi et al. [38] and a Landau-de Gennes mesoscopic approach. The liquid-crystalline order is essentially melted within the defect cores, resulting in high condensation free energy penalties in BPs. These energy penalties are reduced when NPs are trapped in the defect cores, since the highly energetic LC volume is (at least partially) replaced by the non-singular NP volume, thus, the BP structure becomes stable over wider temperature ranges. At the same time, several studies by means of theoretical modelling and simulations focused on the stability of BPs under exogenic impact [67,68,69]. Nevertheless, up to this point, it was not yet clear the relative importance of nanoparticle size, shape, and coating on triggering the stabilization effect. The first systematic investigation of the NP size influence on the BP stabilization by Dierking et al. [70] suggested that NP with sizes below 100 nm are more efficient; above this size, the stabilization mechanism strongly deteriorates.

**Figure 2:**
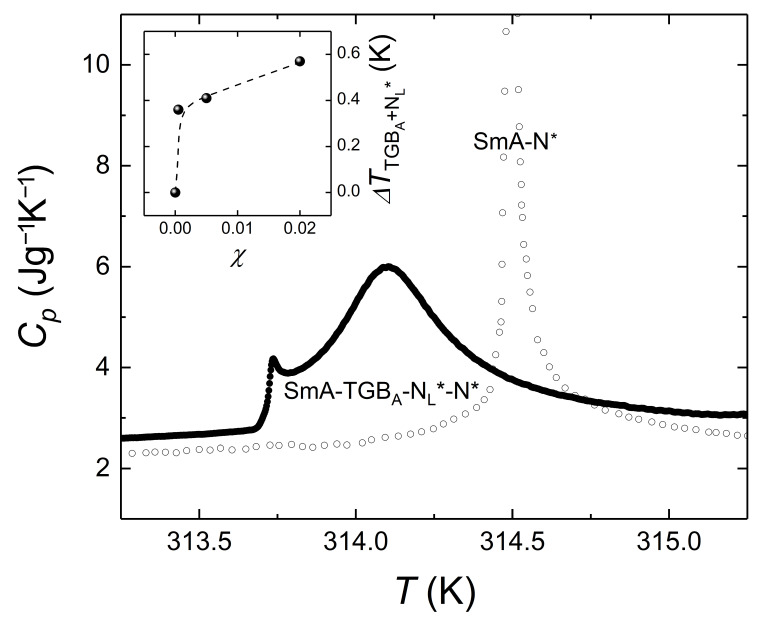
The temperature dependence of heat capacity for pure CE6 (open circles) and its *χ* = 0.0005 mixture of CdSe quantum dots is shown. Pure CE6 shows a sharp, single-peak anomaly denoting a first-order N*-SmA phase transition; on the contrary, the presence of a small concentration of CdSe produces an essentially suppressed, multi-peak anomaly corresponding to the N*-N_L_*-TGB_A_-SmA phase sequence. The inset shows the total range of TGB order, i.e., the N_L_* and TGB_A_ phases, induced in CE6 by CdSe quantum dots [17].

The first report of simultaneous stabilization of two types of defect lines, namely, disclination lines in BPs and screw dislocations in TGBs, came out by Cordoyiannis et al. [17] for OA and TOP-coated CdSe quantum dots dispersed in CE6. BPIII is present in pure CE6 and is ten-fold stabilized for CdSe concentration of *χ* = 0.02 [57]. TGB_A_ phase is absent in pure CE6 and induced by CdSe [17] as depicted by the phase sequence N*-N_L_*-TGB_A_-SmA for *χ* = 0.0005 mixture in Figure 2. Apart from the experimental demonstration of the diverse defect lattices stabilized by CdSe quantum dots [17], an extension of the DCR mechanism [38,66] has been proposed. In particular, for enabling the DCR mechanism, NPs should be effectively driven towards the defect cores. This is favorable only when NPs perturb mildly the surrounding LC order; on the contrary, stronger interactions could trigger phase separation phenomena and degrade the stabilization effect. The resulting perturbations should enable long-range attraction forces driving the NPs towards the defect cores. Hence, the focus of the new mechanism has been on the NPs’ adaptive character. The latter is associated with the NPs’ surface functionalization by flexible molecules that only weakly affect the surrounding LC ordering. This ensures that the free energy gain from replacing the energy-costly defect cores by NPs is not wasted by the disruption of the surrounding LC ordering. The broader mechanism is referred to as the adaptive defect core targeting (ADCT) mechanism [17].

Indispensable additional measurements have been carried out to investigate the simultaneous occurrence of NP-driven BP and TGB stabilization in other soft nanocomposites, as suggested by the ADCT mechanism [17]. In particular, CdSSe quantum dots and spherical Au NPs have been dispersed in CE8. CdSSe quantum dots have essentially the same size as CdSe (diameter of 3.4 nm for CdSSe versus 3.5 nm for CdSe). However, the partial replacement of Se by S resulted in reduced core density and increased surface coverage by OA at the expense of TOP (OA binds on both Cd and S, whereas TOP prefers to bind on Se). Au NPs have a larger core density, a diameter of 10 nm, and OA coating. Hence, CE8 + CdSSe and CE8 + Au mixtures exhibit essential differences compared to CE8 + CdSe ones used in the initial studies [17,56,57], thus, offering testing ground for the stabilization effect and the verification of the ADCT mechanism.

**Figure 3:**
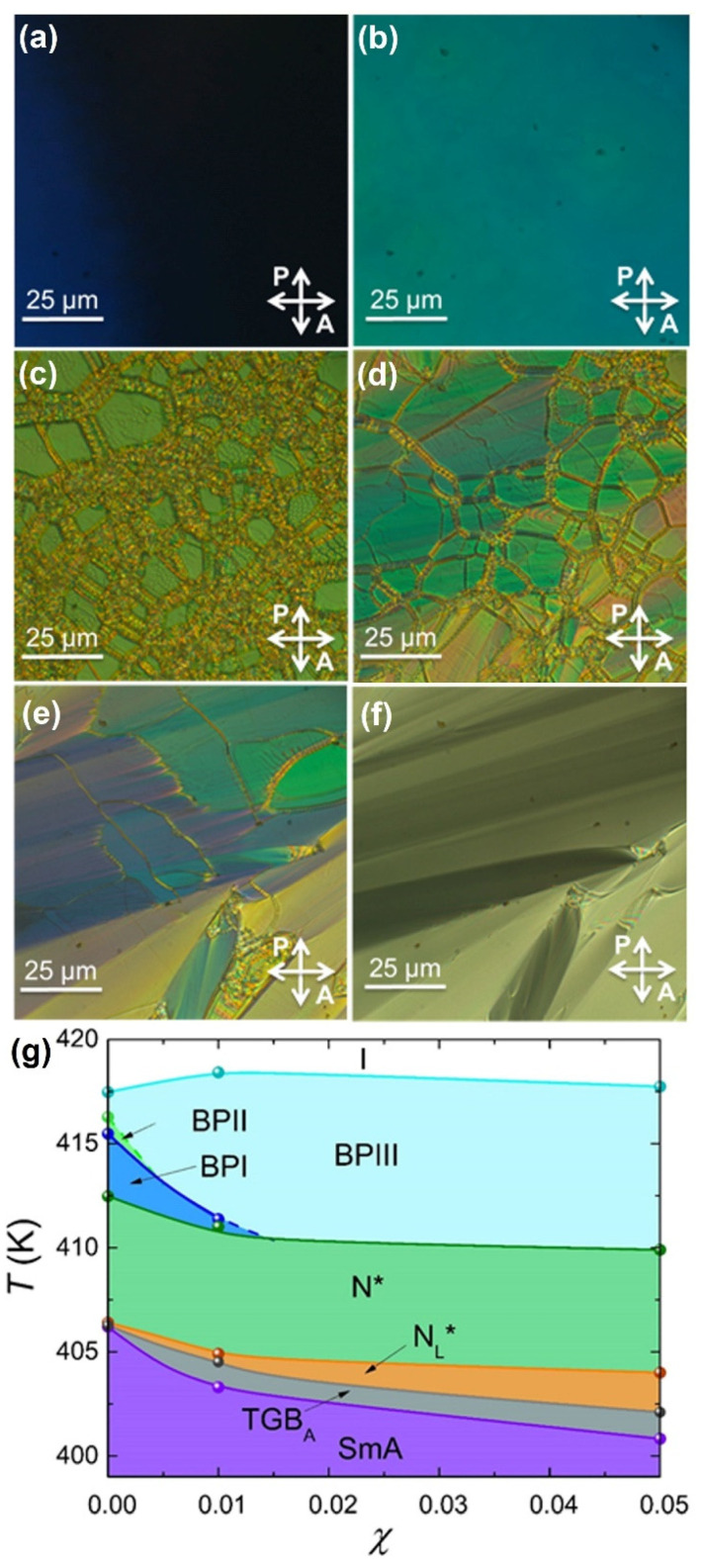
The images show the characteristic textures of the BPIII onset (**a**), BPI (**b**), N* (**c**), N_L_* (**d**), TGB_A_ (**e**), and SmA (**f**) phases, captured for the CE8 + CdSSe *χ* = 0.01 mixture in planar cells, under crossed polarizers. The temperature-concentration (*T-χ*) phase diagram for the CE8 + CdSSe system is presented upon cooling (**g**). A strong stabilization of BPIII is evident at higher temperatures, while the TGB_A_ and N_L_* phases are induced at lower temperatures [59,72].

The temperature profiles of heat capacity and the optical textures have been obtained for several mixtures of CE8 + CdSSe and CE8 + Au NPs by ac calorimetry and polarizing optical microscopy, upon heating and cooling. In both cases, the spherical NPs induce a mild increase of the total BP range and a substantial increase of BPIII range, at the expense of BPII and BPI that gradually disappear [71,72]. In addition, both types of NPs induce the N_L_* and TGB_A_ phases [59,71,73]. The characteristic optical textures of the CE8 + CdSSe *χ* = 0.002 mixture are shown in Figure 3a–f, accompanied by the phase diagram in Figure 3g.

**Figure 4:**
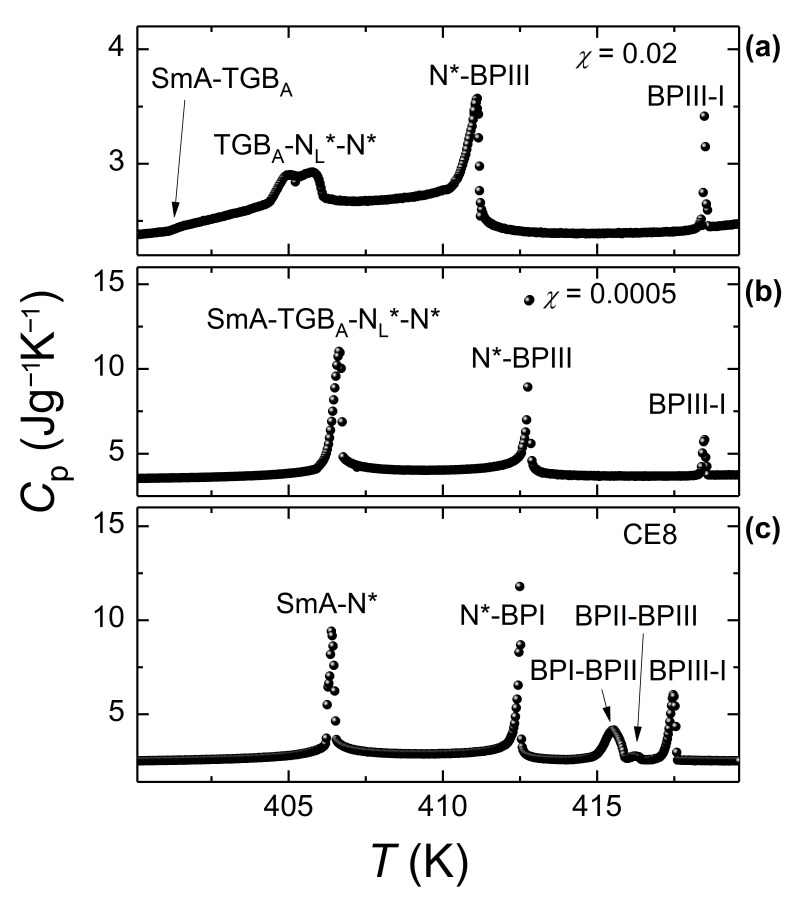
The temperature profiles of heat capacity *C_p_* for pure CE8 (**c**) [56], and two of its mixtures with Au NPs, *χ* = 0.0005 (**b**) [71] and *χ* = 0.02 (**a**), are shown upon cooling from the isotropic down to the SmA phase (rate of 0.25 Kh^−1^).

**Figure 5:**
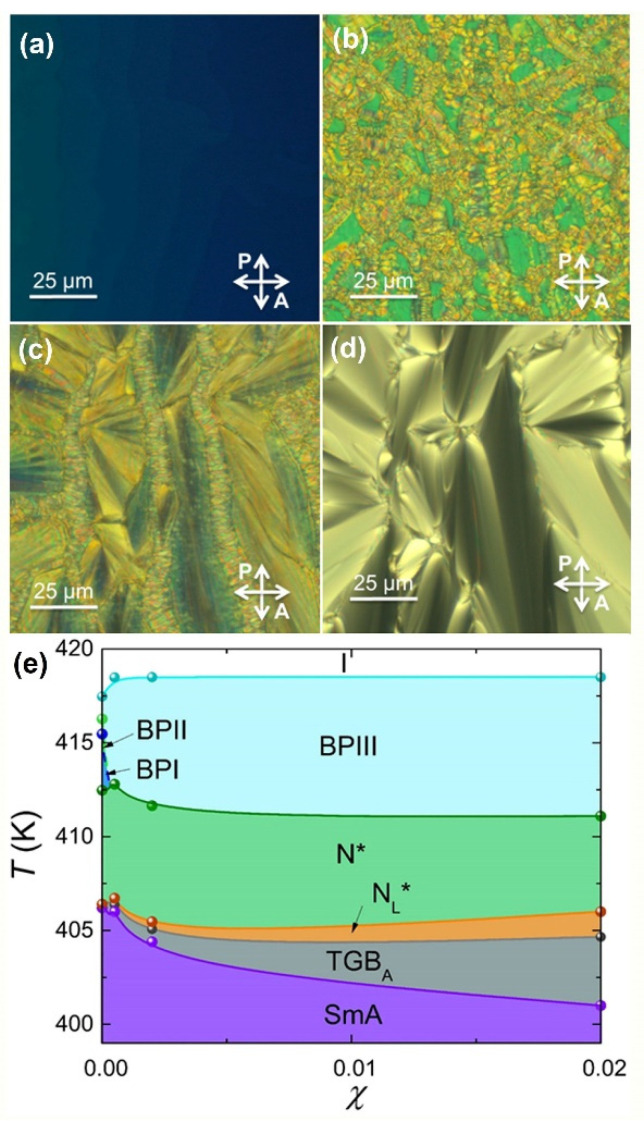
The images show the characteristic textures of the BPIII (**a**), N* (**b**), N_L_*/TGB_A_ coexistence (**c**), and SmA (**d**) phases, captured for the CE8 + Au *χ* = 0.002 mixture in planar cells, under crossed polarizers. The temperature-concentration (*T-χ*) phase diagram for the CE8 + Au system is presented upon cooling (**e**). A strong stabilization of BPIII is evident at higher temperatures, while the TGB_A_ and N_L_* phases are induced at lower temperatures [71,73].

An example of the evolution of heat capacity profiles as a function of Au NPs concentration upon cooling can be seen in Figure 4. Optical textures for the CE8 + Au *χ* = 0.002 mixture are presented in Figure 5a–d, followed by the phase diagram of the CE8 + Au system in Figure 5e.The results on CE8 + CdSSe and CE8 + Au mixtures, indicate that coating of NPs with flexible OA molecules reduces the NP-induced distortions of the surrounding (chiral nematic or smectic) LC ordering, marking the robustness and general character of the ADCT mechanism [17].

### 3.4. Blue Phase and Twist-Grain Boundary Phase Stabilization in Liquid Crystal CE8 Induced by Anisotropic Nanoparticles

The influence of anisotropic NPs, such as nanoplatelets and nanosheets, has been investigated on the stability of BPs and TGBs [74,75,76]. CE8 was chosen as the LC host, to directly compare the results with the ones obtained in the case of spherical NPs. The effect of the core composition, size, and coating has been assessed by choosing 10 nm large MoS_2_ nanoplatelets, as well as 50 nm large r-GO and CoPt-decorated r-GO nanosheets. In all cases, OA has been chosen as the capping agent. The difference between r-GO and CoPt-decorated r-GO is that the latter are heavier and have a higher coverage by OA that binds on the surfaces of both graphene and CoPt. The higher surface coverage in the composite materials is due to the higher affinity of the amine groups to interact with the bimetallic CoPt NPs. Calorimetry and microscopy have been combined to precisely determine the phase sequence and the temperature ranges.

**Figure 6:**
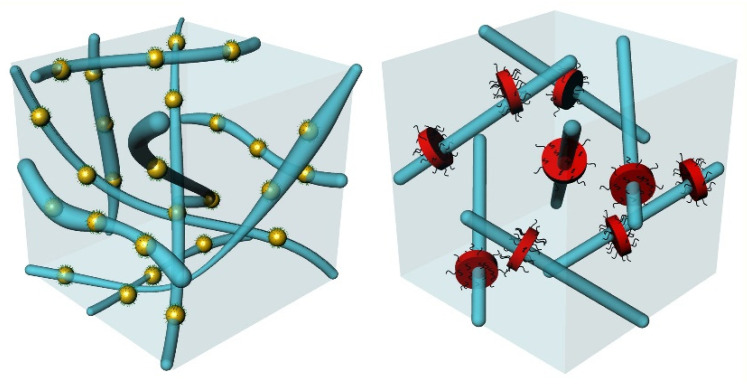
The trapping of isotropic (spherical) and anisotropic NPs is schematically depicted here; the scale and the relative dimensions are approximate. **Left panel**: assembly of spherical NPs along the amorphous lattice of disclination lines in BPIII; **right panel**: assembly of nanosheets along the cubic lattice of disclination lines in BPI.

The anisotropic NPs induce a mild increase of the total BP range of CE8. Contrary to the case of spherical NPs and quantum dots that mostly stabilize BPIII, nanoplatelets and nanosheets stabilize BPI [74,75,76]. The difference is attributed to wetting effects along the large surface of these flat NPs that apparently induce some partial LC order at temperatures inside the isotropic phase of CE8. Wetting effects are the reason behind the strong upshift persistently observed for the phase transition from the isotropic to the liquid-crystalline state (I-BPIII or I-BPI). This upshift is much stronger for large anisotropic NPs with respect to their spherical counterparts dispersed in CE8. Substantial upshift of the isotropic to the liquid-crystalline phase transition has also been reported in other studies of anisotropic NPs (e.g., laponite clay nanoplatelets, graphene oxide nanosheets, carbon nanotubes) dispersed in chiral and non-chiral LCs [77,78,79,80,81,82]. A mean-field-based interpretation has been proposed by Gorkunov and Osipov [83]. The LC order induced in the vicinity of nanoplatelets and nanosheets evidently favors the formation of BPI’s more regular and ordered structure over the macroscopically amorphous BPIII. A simple schematic representation of the NPs trapping within the amorphous and cubic lattices of disclination lines of BPIII and BPI, respectively, can be found in Figure 6.

**Figure 7:**
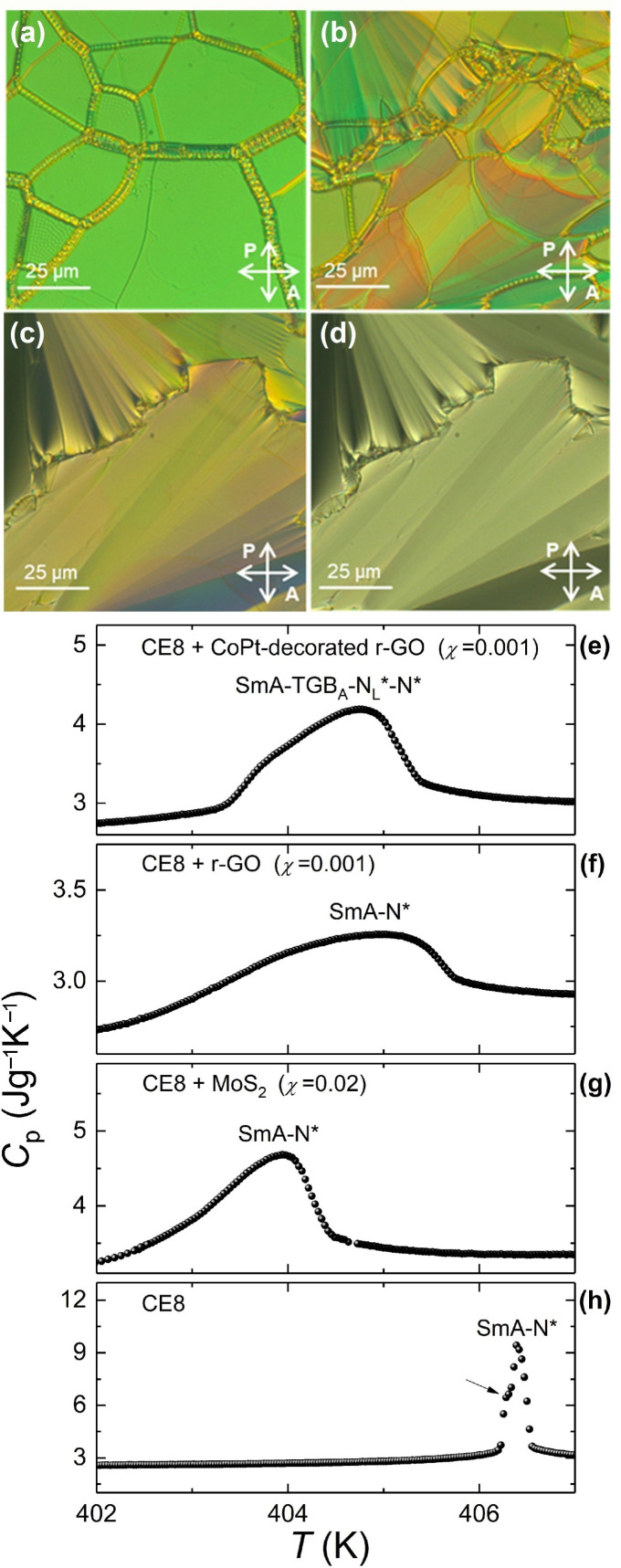
The influence of different types of anisotropic NPs on the N*-SmA phase transition of CE8 is demonstrated. The images show the characteristic textures of the N* (**a**), N_L_* (**b**), TGB_A_ (**c**), and SmA (**d**) phases for the CE8 + CoPt-decorated r-GO *χ* = 0.001 mixture, captured in planar cells under crossed polarizers. The temperature profiles of heat capacity are shown for CE8 + CoPt-decorated r-GO *χ* = 0.001 (**e**), CE8 + r-GO *χ* = 0.001 (**f**), CE8 + MoS_2_ *χ* = 0.02 (**g**) and pure CE8 (**h**) [59]. In all cases, the N*-SmA phase transition is broadened, but only in the case of CE8 + CoPt-decorated r-GO does the phase sequence N*-N_L_*-TGB_A_-SmA appear.

The TGB stabilization exhibits a more complex behavior in the case of large anisotropic NPs. The N*-SmA phase transition of CE8 is suppressed and shifted to lower temperatures as depicted in Figure 7; the arrow in part (**h**) denotes the small shoulder at the low-temperature wing of the heat capacity anomaly indicating the potential presence of a metastable TGB_A_ order in CE8. The NP-induced N*-N_L_*-TGB_A_-SmA sequence is evinced only in the case of CE8 + CoPt-decorated r-GO mixtures [76]; for CE8 + MoS_2_ [75] and CE8 + r-GO [74] no stable TGB_A_ order could be detected by ac calorimetry or optical microscopy. A remarkable difference exists between the same concentration (*χ* = 0.001) of CoPt-decorated r-GO and r-GO. The larger core density and the additional OA on the surface of the nanosheets result in more efficient trapping of the CoPt-decorated r-GO in the screw dislocations and induce a TGB order, as confirmed by both ac calorimetry and microscopy. On the contrary, the r-GO with lower OA coverage is less adaptive to the defect lattice and more strongly disturbs the LC order, as indicated by the significantly suppressed and broadened N*-SmA transition (for a comparison, see the heat capacity profiles in parts (**e**) and (**f**) of Figure 7).

We hereby provide more detailed evidence regarding the broad anomaly attributed to the N*-N_L_*-TGB_A_-SmA sequence in the case of CoPt-coated r-GO. The conventional mode of operation of ac calorimetry has been used in our experiments. This mode renders with precision the transition temperatures. However, it does not sense the total enthalpy change related to a phase transition, but mostly its continuous part. The phase difference between the ac power and sample temperature oscillations is also monitored and provides additional information for the order of the transitions and the coexistence regions (when present). For a continuous (2nd order) phase transition, the temperature profile of phase shift tanφ is inversely proportional to the one of the heat capacity *C_p_*, and the latter has only a real part. For a discontinuous (1st order) phase transition, tanφ shows an anomalous behavior along the phase coexistence region, since *C_p_* has a real and an imaginary part (related to the latent heat). When the precise enthalpy content is of major importance, additional measurements are performed using the relaxation mode that senses the total enthalpy, i.e., its continuous and discontinuous (latent heat) parts. By comparing the results of the two modes of operation, the latent heat of a 1st order transition can be derived. Relaxation runs have not been performed in this case, since such an analysis of the enthalpy content is out of the scope of this work.

**Figure 8:**
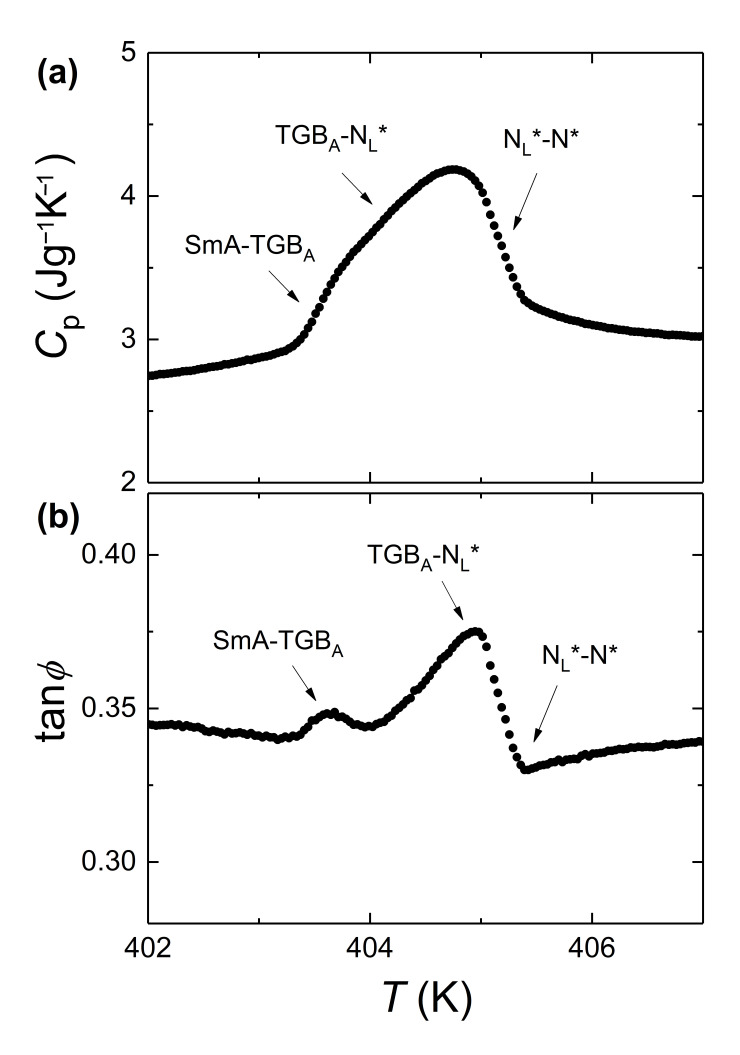
The temperature profiles of *C_p_* (**a**) and tanφ (**b**) are shown for the χ = 0.001 mixture of CE8 + CoPt-decorated r-GO. The data are obtained upon cooling with a scanning rate of 0.2 Kh^−1^.

In Figure 8, both *C_p_* and tanφ are commonly plotted for the CE8 + CoPt-coated r-GO χ = 0.001 mixture. The sharp rise of *C_p_* at the higher temperature side of the peak is related to the onset of N_L_* order. The sharp drop at the lower temperature side is attributed to the onset of SmA order, and the anomalous tanφ behavior (positive spike) suggests a first order TGB_A_-SmA transition. The wider part of the peak is signaling the continuous conversion of N_L_* to TGB_A_ along a broad coexistence region marked by the anomalous behavior of tanφ. The optical textures of all four phases, presented in Figure 7 for this sample, support our conjecture from the calorimetric data for a N*-N_L_*-TGB_A_-SmA sequence. On the contrary, neither N_L_* nor TGB_A_ textures have been detected in the case of r-GO nanosheets and MoS_2_ nanoplatelets. Instead, a coexistence of the oily streaks of N* and the focal conics of SmA phases was persistently present on heating and cooling.

### 3.5. Other Studies on Nanoparticle-Driven Stabilization of Blue Phases and Twist-Grain Boundary Phases

The strategy of dispersing spherical NPs in chiral LCs has been widely adopted regarding BP stabilization over the last ten years. Spherical NPs and colloids in a broad range of sizes (from the smallest NPs of 2 nm to the largest colloids of 1.5 μm) and cores composed of Au, MnO_2_, ZhS, BaTiO_3_, CdSe, PbS, Fe_3_O_4_, SiO_2_, and Ni, have been exploited as stabilization agents [58,84,85,86,87,88,89,90,91,92,93,94]. It is rather impossible to directly compare all these studies because of: (a) essentially different LC hosts (ranging from pure compounds to LC mixtures of variable chemical compositions), (b) NP core composition, size and coating, and (c) different methodologies. Occasionally, the authors refer to the total BP range without identifying which phase has been mostly affected [86,87,89]. Many studies have focused on LC compounds exhibiting only BPII and BPI since the optical textures (colored platelets) and lattice parameters (such as Kossel diagrams [95]) are more straightforward for these phases. On the other hand, BPIII exhibits a weak birefringence, and its optical texture consists of a foggy dark blue color, frequently not trivial to detect by means of optical microscopy (note that under crossed polarizers, the texture of isotropic phase appears black). Nevertheless, some general trends regarding the stabilization effect can be reliably derived. Small size spherical NPs and quantum dots persistently induce an increase of the total BP range. The NP size-dependence studies of Sharma et al. [85] (sizes from 2.2 to 13.2 nm) and Dierking et al. [70] (sizes from 40 nm to 1.5 μm) suggest that, in general, the smaller the NP size, the more efficient the BP stabilization. Small NPs readily assemble at the cores of defects and enable the conditions of DCR and ADCT mechanisms.

Theory predicts [69] phase diagrams with simultaneous stabilization of BPII and BPI for NP sizes up to 100 nm. To the best of the authors’ knowledge, there is only one experimental study supporting the existence of such a phase diagram (an increase of both BPII and BPI ranges) induced by 50 nm large Ni NPs [92]. A substantially different picture is obtained for LCs exhibiting all three BPs, such as CE8 and CE6. Our systematic studies in mixtures of CE6 + CdSe (3.5 nm, OA and TOP coating) [57], CE8 + CdSe (3.5 nm, OA and TOP coating) [56], CE8 + CdSSe (3.4 nm, enhanced OA and reduced TOP coating) [72], and CE8 + Au (10 nm, OA coating) [73] yield phase diagrams resembling to some extent the ones of increased chirality [33,34]. BPIII is enhanced, BPII vanishes, whereas BPI is either mildly affected or gradually disappears under the presence of spherical NPs. Note that the stabilization of all three BPs is of high interest since, apart from the regular lattices of BPII and BPI, the macroscopically amorphous lattice of BPIII also exhibits interesting electro-optical switching properties [30,96,97,98].

The dispersion of anisotropic NPs in LCs for stabilizing BPs shows more consistent results than their spherical counterparts. Regardless of their core composition (e.g., MoS_2_, laponite, graphene oxide, r-GO), size and surface functionalization, nanoplatelets and nanosheets steadily stabilize BPI [74,75,76,78,79,82]. In particular, in the case of r-GO, the stabilization effect is observed only at very small concentrations; at larger concentrations, the stabilization effect is decaying [79,82]. The possible ways that r-GO nanosheets accommodate within the disclination lines have been addressed by Lavrič et al. [76], considering a stacking along or a triangular configuration around the disclination lines. Further simulation studies could assist in revealing the energetically favored configurations as a function of size and anchoring. In a recent study, Draude et al. [99] have reported BP stabilization by means of substantially larger graphene oxide sheets, in the range of few μm. Sheets of such a large size are likely to assemble in the boundaries between BP platelet domains.

The use of other types of anisotropic NPs, such as nanorods, as stabilization agents is studied to a lesser extent. The idea that flexible carbon nanotubes could fill the disclination lines has been explored; however, it was proven rather ineffective with respect to spherical NPs in the initial studies [70]. Recently, the addition of CdS/CdSe nanorods has been reported to increase two-fold the BP range of LC host [93]. The effect has been larger for nanorods than for quantum dots, suggesting that the influence of nanorods is worth more attention. Moreover, the influence of Al_2_O_3_ nanorods on the BP range has been investigated, yielding a maximum stabilization for concentrations around 0.5 wt.% [94]. These recent results suggest that nanorods promote the stabilization of BPII and BPI since their shape matches the geometry of disclination lines. Hybrid stabilization techniques such as the simultaneous use of NPs and polymers have been also tested [70,100,101]. However, hardly any remarkable accumulative effects have been harvested.

The spherical NP-driven stabilization of TGB phases has been studied to a lesser extent compared to BPs. It has been shown in the previous section that the N*-N_L_*-TGB_A_-SmA phase sequence is induced in CE6 + CdSe (3.5 nm, OA and TOP coating) [17], CE8 + CdSSe (3.4 nm, enhanced OA and reduced TOP coating) [59], and CE8 + Au (10 nm, OA coating) [73] mixtures. The stabilization is enabled by the DCR and ADCT mechanisms, i.e., by (a) the partial replacement of the volume of screw dislocations with NPs and (b) the appropriate surface treatment that minimizes the perturbation of the adjacent SmA slabs.

For anisotropic NPs, it has been shown that MoS_2_ nanoplatelets and r-GO nanosheets fail to stabilize the TGB order in CE8. Only CoPt-decorated r-GO renders the N*-N_L_*-TGB_A_-SmA phase sequence [74,75,76]. The nanosheets’ increased density and enhanced OA coverage (for CoPt-decorated r-GO) likely hold the key role behind the difference between two types of r-GO nanosheets. The results of spherical NPs also support this hypothesis. In particular, when comparing the temperature range of TGB order (N_L_* and TGB_A_ phases) of CE8 + CdSSe and CE8 + Au mixtures [59,73], the stabilization effect is larger in the case of Au NPs. The Au NPs with heavier core (than CdSSe) apparently sense a less viscous LC medium and, therefore, they assemble in the cores of defects more efficiently. Moreover, the OA coating of Au NPs makes them adaptive to the surrounding LC order regardless of their larger size.

**Figure 9:**
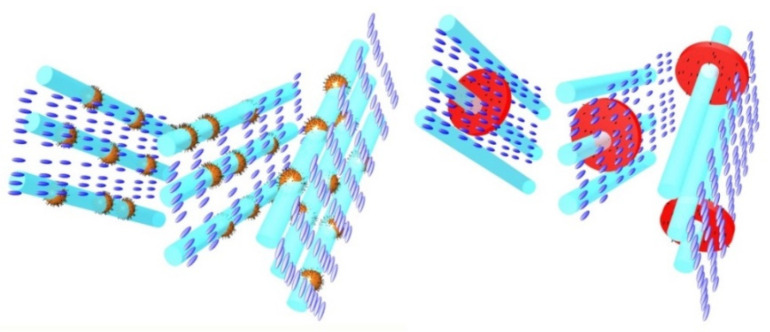
The assembly of spherical (**left panel**) and anisotropic NPs (**right panel**) along the lines of screw dislocations in the TGB_A_ phase is visualized; the scale and the relative dimensions are approximate. Our results suggest a stacking assembly of nanosheets along the screw dislocations.

In Figure 9, the assembly of spherical and anisotropic NPs in the grain boundaries is schematically depicted. NPs assemble within the lines of screw dislocations along the grain boundaries. Though the picture is straightforward for spherical NPs, in the case of graphene nanosheets, one could assume different configurations, such as stacking along or parallel to the screw dislocations (along the walls of grain boundaries). Using small-angle X-ray scattering it has been shown that an increased SmA layer periodicity is sensed in the case of CE8 + CoPt-decorated r-GO mixture of *χ* = 0.001 [102]. This result is in favor of the assembly of CoPt-decorated r-GO between the SmA layers. Based on the above, we anticipate that the nanosheets assemble perpendicularly to the direction of the lines of screw dislocations in TGB_A_. Upon further cooling, the mixture into the SmA phase, the grain boundaries with screw dislocations disappear, and the nanosheets remain between the smectic layers. Nevertheless, for essentially larger sheets sizes, such as the ones in μm scale used in other studies [99], a preferable assembly along the surfaces of the grain boundaries cannot be excluded. Regarding using other types of NP geometries, a recent study by Sahoo et al. [103] reports the TGB_C_* phase stabilization by a small concentration of dispersed Au nanorods.

The NP-driven BP stabilization gives an apparently milder effect compared to the case of polymers. However, most of the studies are based only on optical measurements, lacking detailed information about thermal history and possible appearance of super-cooling phenomena [62] in these first order transitions. Therefore, a direct comparison of the stabilization effect of different strategies (polymers, NPs, dopants) is ambiguous, unless the results are obtained by the same methodology and on the same LC host. A clear advantage of NP-driven BP stabilization is that by selecting appropriate types of NPs, one can tune the electro-optical properties (Kerr effect, driving voltage) of the nanocomposite, as demonstrated for BaTiO_3_ [58] and graphene nanosheets [104]. In the case of TGBs, the effect of Au nanorods on the LC material’s structural and photonic bandgap has also been reported [103]. The optimization of the aforementioned properties is of major importance towards potential applications in display technologies.

## 4. Conclusions and Prospects

An overview of the recent advances in NP-driven BP and TGB phase stabilization has been provided, focusing on experimental studies. Calorimetric and optical measurements, as well as the resulting phase diagrams, have been presented on BP and TGB stabilization by: (a) spherical CdSe, CdSSe, and Au NPs [17,56,57,59,71,72,73], and (b) MoS_2_ nanoplatelets, r-GO and CoPt-decorated r-GO nanosheets [74,75,76]. The simultaneous use of calorimetry and microscopy confirms the thermodynamic stability of these phases. This is of major importance since, as reported in several studies, LC phases (especially the ones associated with 1st order transitions) can be super-cooled [62] or apparently induced in thin samples by the interfaces [60,93]. The outcome of our studies and other studies in literature can be summarized as follows. First, smaller size NPs (below 100 nm) are more easily trapped in the cores of defects. Second, spherical NPs tend to stabilize more BPIII, while nanoplatelets and nanosheets favor BPI when dispersed in LC compounds where all three BPs exist. Third, spherical NPs appear more effective towards the TGB stabilization than their anisotropic counterparts. Fourth, a higher core density and an enhanced surface coating of NPs with long flexible chains (such as OA) are propitious for stabilization. Moreover, recent studies suggest that the existing mechanisms (DCR and ADCT) could be further improved by focusing on the importance of saddle-splay elasticity and Gaussian curvature [71,105,106].

The study of NP-driven stabilization of BPs and TGBs is of multifold importance. From a fundamental point of view, it contributes to a deeper understanding of the conditions needed for the trapping of inclusions in defect lattices and the development of theoretical mechanisms, such as the DCR and ADCT [17,38,66]. For envisioned technological applications, the controlled trapping of NPs in regular arrays can open up new avenues towards soft nanocomposites with exceptional properties. Typical such examples are three-dimensional, BP-based photonic crystals [107,108], external-field-controlled soft materials [109], and multi-ferroics [110], as well as tunable metamaterials [111]. Consequently, the dispersion of NPs in LCs attracts a constantly increasing interest, both experimental and theoretical.

Over the last ten years, great experimental progress has been made with templating NPs and colloids in regular arrays by exploiting the defect lattices of soft liquid-crystalline matrices [14,17,18,19,20]. This generates an emerging need for complemental molecular simulations in order to uncover the favorable NP configurations along the cores of defects. At the same time, not only do NPs trapped within LC lattices increase the stability range of certain liquid-crystalline phases, but they also selectively tune the photonic bandgap of the LC hosts [36,103]. Thus, we anticipate that the ongoing research on NP-driven stabilization of LC phases, through the assembly of the former in the defect lattices of the latter, will provide further insight into aspects in fundamental physics and lead to novel technological applications.

## Data Availability

Calorimetric raw data and images from microscopy textures can be provided after reasonable requests to the corresponding author.
